# Comparison of a Single Assessment Numeric Evaluation Method and the Banff Patella Instability Instrument 2.0 for Patients With Patellofemoral Instability

**DOI:** 10.1177/23259671241265836

**Published:** 2024-10-01

**Authors:** Sebastian Gebhardt, Julian Flügel, Georgi I. Wassilew, Peter Balcarek

**Affiliations:** † Center for Orthopaedics, Trauma Surgery and Rehabilitation Medicine, University Medicine Greifswald, Greifswald, Germany; ‡ ARCUS Sportklinik, Pforzheim, Germany; Investigation performed at ARCUS Sportklinik, Pforzheim, Germany

**Keywords:** knee, patella, patellofemoral instability, patient-reported outcome measures, single assessment numeric evaluation

## Abstract

**Background:**

The concept of Single Assessment Numeric Evaluation (SANE) has been introduced for several clinical entities; however, a validated SANE focusing on patients with patellofemoral instability has not been described.

**Purpose/Hypothesis:**

The purpose of this study was to investigate the expressiveness of SANE questions (SQs) for the assessment of patients experiencing patellofemoral instability. It was hypothesized that the complexity of patellofemoral instability cannot be demonstrated by a single question.

**Study Design:**

Cohort study (diagnosis); Level of evidence, 2.

**Methods:**

Between October 2022 and March 2023, 120 consecutive patients (male/female, 50/70; mean age, 23.9 ± 8.0 years; mean body mass index, 25.3 ± 5.1 kg/m^2^) with patellofemoral instability were assessed with the Banff Patella Instability Instrument 2.0 (BPII 2.0). Patients were randomized into 3 groups (40 patients each) and asked to answer 1 of 3 SQs: “How do you rate your knee joint if a completely stable kneecap means 100%?” (SQ 1), “How do you rate your knee joint if complete satisfaction means 100%?” (SQ 2), and “How do you rate your knee joint if complete normal function means 100%?” (SQ 3). Means ± standard deviations were compared using 1-way analysis of variance, the correlation between BPII 2.0 and each SQ was assessed by Pearson correlation, and Bland-Altmann analysis was performed to investigate biases of each SQ in comparison with BPII 2.0.

**Results:**

The mean BPII 2.0 score was 40.5 ± 16.8 points, and the mean results of SQ 1, SQ 2, and SQ 3 were 44.2% ± 26.0%, 42.6% ± 25.4%, and 44.2% ± 18.9%, respectively, without significant differences between the groups (all *P* > .05). The correlations between BPII 2.0 and SQ 1, SQ 2, and SQ 3 were high (*r* = 0.75; 95% CI, 0.58 to 0.86; *P* < .0001), low (*r* = 0.35; 95% CI, –0.05 to 0.6; *P* = .02), and low (*r* = 0.31; 95% CI, –0.002 to 0.56; *P* = .051), respectively. Bland-Altman analysis between BPII 2.0 and SQ 1, SQ 2, and SQ 3 revealed biases of −0.12 (SD, 17.1), –1.45 (SD, 24.4), and −8.0 (SD, 19.4), respectively.

**Conclusion:**

The SQ “How do you rate your knee joint if a completely stable kneecap means 100%?” demonstrated concurrent validity with the BPII 2.0 and may serve as a helpful tool to quickly assess patients with recurrent patellofemoral instability in a preoperative setting.

The incidence of first-time patellar dislocation is reported to be 5.8 to 77 per 100,000 persons, with a risk of recurrent dislocation in approximately half of cases.^1,5,12,21^ Thus, patellofemoral instability is a significant problem leading to pain, loss of function, and subsequently reduced quality of life for affected patients.^8,16^

A prerequisite for the measurement of the individual disease burden as well as the development and improvement of evidence-based treatment methods is validated outcome measurement. For this purpose, patient-reported outcome measures (PROMs) have moved into focus in recent years.^11^ As suitable PROMs must be patient group and injury specific, the Banff Patella Instability Instrument 2.0 (BPII 2.0) was developed to specifically evaluate patients with patellar instability.^10^ The BPII 2.0 is a 23-item score that has been tested for validity and reliability^14^ and has furthermore been translated and validated in German.^3^ However, BPII 2.0 has certain disadvantages in common with other multi-item PROMs; because of their length, missing data are a common source of bias of different established PROMs.^7,17,19^ Furthermore, impairments of the adjacent joints or the contralateral leg and inapplicable questions can influence the results of PROMs, a problem that has been specifically reported for BPII 2.0 before.^10^

To address these shortcomings and enable a more efficient way to evaluate patient-specific outcomes, Single Assessment Numeric Evaluation (SANE) by a single question has been introduced.^23^ Different SANE questions (SQs) have been established for patients experiencing osteoarthritis, focal cartilage lesions, and ligament and meniscal injury of the knee and have been validated in comparison with the International Knee Documentation Committee questionnaire, the Knee injury and Osteoarthritis Outcome Score, and the Lysholm score.^6,18^ However, to date, no SQ specifically focusing on patellofemoral instability has been established. In the present study, we therefore sought to investigate the expressiveness of 3 different SQs in comparison with BPII 2.0 for the assessment of patients experiencing patellofemoral instability. We hypothesized that the complexity of patellofemoral instability cannot be sufficiently demonstrated by a single question.

## Methods

Before enrolling patients in this prospective study, approval of the local ethics committee was obtained (F-2019-070), and every included patient signed a written informed consent form to participate.

Between October 2022 and March 2023, 120 consecutive patients (male/female, 50/70; mean age, 23.9 ± 8.0 years; mean body mass index, 25.3 ± 5.1 kg/m^2^) with patellofemoral instability presenting to a specialist consultation for joint-preserving knee surgery in a privately run orthopaedic clinic were included in the study. The prerequisite for participation was recurrent patellar instability with at least 2 previous dislocations. Previous patellar stabilizing surgeries, osteoarthritis (Kellgren-Lawrence grade >2), rheumatoid arthritis, infection, and concomitant injuries such as ligament injury or meniscal injury were reasons for exclusion. All patients included in the study presented at a minimum time interval of 4 weeks after the last patellar dislocation to plan a therapeutic procedure. Because the center at which the investigation was performed does not have an emergency department, patients with acute dislocations were not seen in the outpatient clinic. All surveys took place before a possible surgical intervention.

All 120 patients completed the German version of the BPII 2.0.^3^ The BPII 2.0 is a validated PROM consisting of 23 items grouped into 5 sections (Symptoms and Physical Complaints, Work and/or School Related Concerns, Recreation/Sport/Activity, Lifestyle, and Social and Emotional).^13^ Patients were asked to rate each of the 23 items on a scale from 0 to 100. The overall BPII 2.0 score is the mean of all 23 items. A BPII 2.0 score of 0 indicates severe problems, and a BPII 2.0 score of 100 indicates no problems with patellofemoral instability of the knee.

Additionally, patients were randomized into 1 of 3 groups containing 40 patients each. Three possible SQs were created in consensus and evaluated to test for their suitability to assess patients with patellofemoral instability. This approach has previously been described as adequate to achieve content validity.^4^ For all questions, patients could choose a numeric value between 0% and 100% to evaluate the state of their knee, where 0% indicated severe problems and 100% indicated no problems with the knee. SQ 1 was, “How do you rate your knee joint if a completely stable kneecap means 100%?” SQ 2 was, “How do you rate your knee joint if complete satisfaction means 100%?” SQ 3 was, “How do you rate your knee joint if complete normal function means 100%?” Patients were asked to answer SQ 1, SQ 2, or SQ 3 in addition to BPII 2.0.

Statistical analysis was carried out by Prism Version 9.5.0 (GraphPad Software). Data were tested for normality by the Shapiro-Wilk test and are displayed as means ± standard deviations. One-way analysis of variance with a Bonferroni multiple comparison test was used to test for significance between the groups. The level of significance was set at a *P* value of <.05. The correlation between the BPII 2.0 and the SQs was analyzed with the Pearson correlation coefficient (*r*) with a 95% CI. Bland-Altman analysis (plots) was used to investigate biases (differences between the means) of each SQ in comparison with BPII 2.0.

Correlation strength was defined as very high if the *r* value was above 0.90, high if the *r* value was between 0.70 and 0.89, moderate if the *r* value was between 0.50 and 0.69, low if the *r* value was between 0.30 and 0.49, and negligible if the *r* value was below 0.30.

## Results

Demographic data are displayed in Table 1, and no differences between the groups were observed (all *P* > .05).

**Table 1:** Patient Demographic Data in the Groupwise Comparison^*a*^

	SQ 1	SQ 2	SQ 3	*P*
Female/male ratio	20/20	22/18	28/12	.17
Age, y	23.4 ± 7.2	22.3 ± 6.5	25.7 ± 9.5	.14
BMI, kg/m^2^	25.6 ± 5.2	24.7 ± 4.9	25.6 ± 5.3	.68

a Values are presented as mean ± SD unless otherwise indicated. BMI, body mass index; SQ, Single Assessment Numeric Evaluation question.

**Figure 1.**
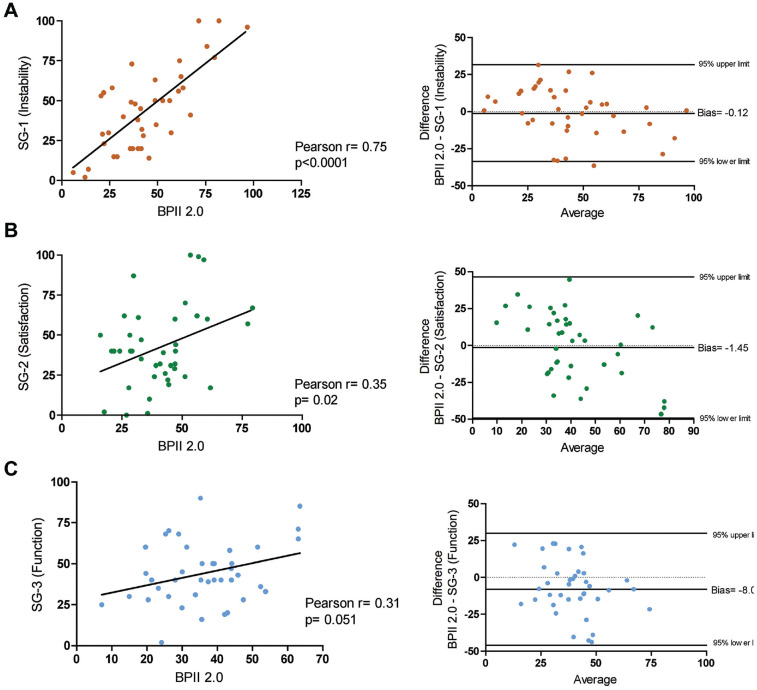
Pearson correlation and Bland-Altmann plots of (A) SQ 1, (B) SQ 2, and (C) SQ 3 in comparison with the Banff Patella Instability Instrument 2.0 (BPII 2.0). SQ, SANE question.

The mean BPII 2.0 score of the total cohort was 40.5 ± 16.8 points, and the mean values for the SQs were 44.2% ± 26.0% for SQ 1, 42.6% ± 25.4% for SQ 2, and 44.2% ± 18.9% for SQ 3, with no difference between SQ 1, SQ 2, and SQ 3 (all *P* > .05). The correlation was high between BPII 2.0 and SQ 1 (*r* = 0.75; 95% CI, 0.58 to 0.86; *P* < .0001), low between BPII 2.0 and SQ 2 (*r* = 0.35; 95% CI, –0.05 to 0.6; *P* = .02), and also low between BPII 2.0 and SQ 3 (*r* = 0.31; 95% CI, –0.002 to 0.56; *P* = .051) (Figure 1).

As revealed by Bland-Altman analysis and illustrated by the Bland-Altman plots (Figure 1), BPII 2.0 in comparison with SQ 1 and SQ 2 showed similar results with calculated biases (difference between the means) of only −0.12 (SD of bias, 17.1) and −1.45 (SD of bias, 24.4), respectively. Analysis of the BPII 2.0 in comparison with SQ 3 revealed a bias of −8.0 (SD of bias, 19.4), indicating that patients rated their knee function on average 8 percentage points inferior to the corresponding BPII 2.0 score value.

## Discussion

The main finding of the present study was that the SQ “How do you rate your knee joint if a completely stable kneecap means 100%?” had a high correlation with the BPII 2.0, and thus concurrent validity for the preoperative evaluation of the disease-specific burden of patellofemoral instability was confirmed.

The BPII, launched in 2013, underwent validation against the Norwich Patellar Instability Score^20^ and Kujala score^10^ for patients with patellofemoral instability, demonstrating a reasonably strong correlation with existing outcome measures.^9^ Subsequently, the BPII underwent factor analysis and item reduction, leading to the development of the current BPII 2.0.^13^ The BPII 2.0 is a disease-specific quality of life assessment composed of 23 questions organized into 5 domains, encompassing symptoms and physical issues; work-related considerations; engagement in sports, recreation, and competition; lifestyle factors; and social and emotional aspects. Research has confirmed the BPII 2.0 is a valid and reliable disease-specific PROM suitable for use in the adolescent population as well.^14^ Despite its specific design for patellar instability and the absence of ceiling effects, utilization of the BPII 2.0 has been limited because of its length and complexity, thus leading to varying response rates and provoking biases.^15^

A valid SQ for patellofemoral instability would avoid these shortcomings. However, because patellofemoral instability is a complex disease pattern that is influenced by a number of anatomic and patient-specific risk factors, we hypothesized that, unlike for knee ligament injuries,^24^ osteoarthritis, or cartilage damage,^22^ the burden of patellofemoral instability cannot adequately be reflected by a single question. However, the results of our study indicate that the results of SQ 1, which focuses on the subjectively perceived stability of the patella, are highly correlated with the results of the much more detailed and complex BPII 2.0. Additionally, SQ 1 provides the advantages of a simple and fast recording compared with BPII 2.0. In contrast, SQ 2 and SQ 3, focusing on satisfaction with the knee joint and function of the knee joint in general, did not correlate with BPII 2.0 to a clinically meaningful extent.

The high correlation of SQ 1 with the BPII 2.0 confirms concurrent validity of SQ 1. Since the BPII 2.0 is a comprehensive and established score for the evaluation of patellofemoral instability, the high correlation supports the assumption that construct validity is also present. However, construct validity must be confirmed by further testing of SQ 1 in connection with different knee injuries. The formulation of SQ 1 in an expert consensus fulfills the requirements for content validity, but it can be also confirmed by discussing SQ 1 in a larger group of experts. To date, SQ 1 cannot replace the BPII 2.0 because, unlike for SQ 1, test-retest reliability and responsiveness to therapeutic interventions have been demonstrated for the BPII 2.0.^14^

The present study closes a gap by introducing the first single numeric evaluation question specifically for patients with patellofemoral instability. In addition to several strengths, such as a large number of included patients and a specifically selected patient group, as well as a valid score for comparison, our study has certain limitations. Concerning the aspects of construct and content validity, further testing and expert evaluation of SQ 1 are required. Because of its design, the study does not allow conclusions on intra- and interobserver reliability. Furthermore, the responsiveness of the investigated SQs in connection with patients experiencing patellofemoral instability after therapeutic interventions has to be investigated. To further assess the objectivity of SQ 1, the evaluation would have to be repeated in a different clinical setting. While SQ 1 is suitable to assess the disease-specific burden of patellofemoral instability in a preoperative setting, the assessment is insufficient to draw conclusions on therapeutic consequences and on postoperative evaluations. The treatment of patellofemoral instability is complex, and decision-making for nonoperative or operative therapy depends on the estimated redislocation risk and exact analysis of the underlying triggering factors, which cannot be replaced by a single question.^2^ However, individual disease-specific psychological distress is a further relevant parameter that should be taken into consideration when deciding on treatment options. In this regard, the SQ may serve as a helpful tool.

## Conclusion

The SQ “How do you rate your knee joint if a completely stable kneecap means 100%?” demonstrated concurrent validity with the BPII 2.0 and may serve as a helpful tool to quickly assess patients with recurrent patellofemoral instability in a preoperative setting.
